# Dental Implant Outcomes in Patients with Cleft Lip, Alveolus and/or Palate: A Systematic Analysis of Clinical Studies

**DOI:** 10.3390/medicina62030569

**Published:** 2026-03-18

**Authors:** Andrei Tent, Alexandru Mester, Armencea Gabriel, Simion Bran, Dacian Sabau, Andra Piciu, Florin Onisor

**Affiliations:** 1 Department of Oral and Maxillo-Facial Surgery, Faculty of Medicine and Pharmacy, University of Oradea, 410087 Oradea, Romania; tent_andrei@yahoo.com (A.T.);; 2 Department of Oral Health, University of Medicine and Pharmacy “Iuliu Hatieganu”, 400012 Cluj-Napoca, Romania; 3 Department of Maxillofacial Surgery and Implantology, University of Medicine and Pharmacy “Iuliu Hatieganu”, 400012 Cluj-Napoca, Romania; 4 Department of Medical Oncology, University of Medicine and Pharmacy “Iuliu Hatieganu”, 400012 Cluj-Napoca, Romania

**Keywords:** cleft lip, cleft palate, cleft alveolus, orofacial cleft, dental implants, osseointegration

## Abstract

*Background and Objectives*: Dental implant placement in grafted alveolar cleft sites has become an integral component of comprehensive cleft rehabilitation. However, survival outcomes vary across studies, and temporal trends in clinical performance have not been systematically quantified. This review aimed to evaluate implant survival in grafted alveolar cleft patients and to compare outcomes between early and modern treatment eras. *Materials and Methods*: A systematic search of the PubMed, Web of Science, Cochrane Library, and Wiley databases was performed in accordance with PRISMA guidelines. Clinical studies reporting implant survival in grafted alveolar cleft sites with a minimum follow-up of 12 months were included. Data extraction encompassed implant survival, timing of placement, grafting protocols, and reported causes of failure. For temporal comparison, studies were stratified into an early era (1997–2008) and a modern era (2010–2026). Weighted pooled survival rates were calculated, and differences between proportions were assessed using a two-proportion Z-test (*p* < 0.05). *Results*: 18 studies met the inclusion criteria, representing 1561 implants placed in grafted alveolar cleft sites. Overall reported survival ranged from 80% to 100%. Weighted pooled survival increased from 91.2% (95% CI: 87.9–94.5) in early studies to 94.2% (95% CI: 92.9–95.5) in modern cohorts, demonstrating a statistically significant 3.0% absolute improvement (*p* = 0.038). Implant failures occurred predominantly during the early osseointegration phase and were commonly associated with insufficient graft volume or inadequate primary stability. Late biological complications were infrequently reported. *Conclusions*: When appropriate bone reconstruction, healing, and multidisciplinary coordination are achieved, implant therapy represents a reliable component of comprehensive cleft care. Further prospective studies with standardized protocols and long-term follow-up are needed to strengthen evidence-based recommendations.

## 1. Introduction

Orofacial clefts, including cleft lip, cleft alveolus, and cleft palate, represent some of the most common congenital craniofacial anomalies worldwide, with an incidence ranging from 1:500 to 1:1000 live births depending on ethnicity and geographic region [1,2]. These conditions frequently involve the alveolar process of the maxilla, leading to discontinuity of the dental arch, altered tooth eruption patterns, and disturbances in maxillofacial growth [2]. Beyond structural impairment, alveolar clefts are associated with significant functional and psychosocial consequences, affecting mastication, speech, facial harmony, and overall quality of life across different stages of development [3].

A characteristic feature of alveolar clefts is the deficiency or absence of bone within the cleft region, which compromises normal dental eruption and orthodontic alignment [4]. Hypodontia, particularly of the maxillary lateral incisors adjacent to the cleft, is common and further complicates restorative planning [3,4]. For this reason, alveolar bone grafting (ABG) constitutes a central component of interdisciplinary cleft management, aiming to restore maxillary continuity, facilitate canine eruption, permit orthodontic tooth movement, and establish a stable osseous foundation for future prosthetic rehabilitation [2]. Secondary alveolar bone grafting is typically performed during the mixed dentition stage, when the maxillary canine root has reached approximately two-thirds of its development [1,5]. Autogenous iliac crest bone remains the gold standard donor material due to its favorable osteogenic, osteoinductive, and osteoconductive properties [6,7].

Despite its established role, ABG is subject to biological remodeling and variable resorption patterns, and not all grafts maintain sufficient volume at skeletal maturity [4,5,6,7,8]. In some cases, residual defects or inadequate ridge dimensions necessitate secondary or tertiary augmentation procedures prior to definitive rehabilitation [4,5,6,7,8]. These factors have direct implications for implant placement, as graft volume, density, and structural stability influence primary implant stability and osseointegration [9].

Dental-implant-supported rehabilitation has become an integral option for replacing missing teeth in patients with cleft lip and/or palate [10]. Compared with conventional fixed or removable prostheses, implant therapy avoids preparation of adjacent teeth, provides functional loading of the reconstructed alveolar ridge, and allows for individualized prosthetic reconstruction of the cleft site [9,10]. However, implant placement in previously grafted regions may be influenced by altered bone morphology, differences in trabecular architecture, and scarred peri-implant soft tissues, potentially affecting surgical planning and prosthetic execution [9].

Although numerous clinical investigations have evaluated dental implant placement in grafted alveolar cleft sites, the available literature demonstrates variability in study design, follow-up duration, grafting protocols, and outcome definitions [8,9,10]. Reported outcomes are often derived from retrospective cohorts, and comprehensive evaluation of temporal trends and failure characteristics remains limited.

The aim of this systematic review is to critically evaluate the existing clinical evidence regarding the survival and success rate of dental implants placed in grafted alveolar cleft regions in patients with cleft lip and/or palate. The review will synthesize all available evidence to evaluate the predictability and limitations of implant-supported rehabilitation in grafted alveolar cleft sites and to identify gaps in the current literature requiring further high-quality research.

## 2. Materials and Methods

### 2.1. Study Design and Reporting Guidelines

This systematic review was conducted in accordance with the Preferred Reporting Items for Systematic Reviews and Meta-Analyses [11]. The review protocol was developed a priori and followed a predefined research question, eligibility criteria, and methodological framework to minimize selection bias and enhance transparency.

The present study was conducted as a systematic review with pooled quantitative analysis of observational studies. Registration in PROSPERO was not performed, as prospective registration is recommended but not required for this type of review. A predefined protocol, eligibility criteria, and statistical plan were established before study initiation and the review was conducted in accordance with PRISMA 2020 guidance.

### 2.2. Focused Question and PICO Framework

The review was structured using the Population, Intervention, Comparison, and Outcome (PICO) framework.

- Population: Patients with congenital alveolar clefts associated with cleft lip and/or palate.
- Intervention: Placement of dental implants in previously grafted alveolar cleft sites.
- Comparison: Not mandatory; when available, comparisons between different grafting protocols, defect morphologies, or implant approaches were considered.
- Outcomes: Primary outcome was dental implant survival. Secondary outcomes included causes of implant loss and reported implant-related complications.

The focused research question was as follows:

What are the survival rates and reported causes of implant loss for dental implants placed in grafted alveolar cleft sites?

### 2.3. Eligibility Criteria

#### 2.3.1. Inclusion Criteria

Studies were included if they met all of the following criteria:

1. Human clinical studies involving patients with alveolar clefts;
2. Alveolar bone grafting performed prior to implant placement;
3. Dental implants placed in the grafted cleft region;
4. Implant survival reported as an outcome;
5. Minimum follow-up of 12 months after implant placement;
6. Prospective or retrospective cohort studies and case series more than 10 patients;
7. Articles published in the English language.

#### 2.3.2. Exclusion Criteria

Studies were excluded if they met any of the following criteria:

- Case reports or case series with fewer than 10 patients;
- Animal, in vitro, finite element, or cadaveric studies;
- Studies focusing exclusively on bone grafting or augmentation without implant survival outcomes;
- Studies reporting short-term osseointegration only, without survival data;
- Mixed populations in which cleft-specific implant outcomes could not be extracted;
- Reviews, editorials, technical notes, or conference abstracts.

### 2.4. Information Sources and Search Strategy

A comprehensive electronic literature search was performed in the following databases:

- PubMed/MEDLINE;
- Web of Science;
- Wiley Online Library.

The search covered all records from database inception to the most recent available publications.

The PubMed search strategy incorporated Medical Subject Headings (MeSH) and Title/Abstract keywords as follows: ((“Cleft Lip”[Mesh] OR “Cleft Palate”[Mesh] OR “cleft lip”[tiab] OR “cleft palate”[tiab] OR “cleft alveolus”[tiab] OR “orofacial cleft*”[tiab]) AND (“Dental Implants”[Mesh] OR “dental implant*”[tiab] OR “oral implant*”[tiab])). Filters were applied to restrict results to human studies and articles published in English.

In Web of Science, the search was performed using the Topic field (TS), which includes title, abstract, author keywords, and Keywords Plus: TS = (“cleft lip” OR “cleft palate” OR “cleft alveolus” OR “orofacial cleft*”) AND TS = (“dental implant*” OR “oral implant*”). No document-type restrictions were initially applied. Results were subsequently screened according to predefined eligibility criteria.

For Wiley Online Library, the search was conducted using keyword fields (title, abstract, and keywords): (“cleft lip” OR “cleft palate” OR “cleft alveolus” OR “orofacial cleft*”) AND (“dental implant*” OR “oral implant*”). Search results were filtered for clinical studies involving human subjects in English language.

Reference lists of included full-text articles were also manually screened to identify additional relevant studies.

### 2.5. Study Selection Process

All records retrieved from the electronic searches were imported into Rayyan systematic review software, where duplicate records were removed. Title and abstract screening were performed initially to exclude clearly irrelevant studies. Full-text articles of potentially eligible studies were then assessed independently against the predefined inclusion and exclusion criteria.

Discrepancies during full-text screening were resolved through discussion and consensus. Reasons for full-text exclusion were documented systematically and are presented in a dedicated exclusion table in accordance with PRISMA recommendations.

### 2.6. Data Extraction

Data extraction was performed using a standardized data extraction form. The following variables were collected from each included study:

- Study design and year of publication;
- Number of patients and implants;
- Type of cleft deformity;
- Grafting technique and augmentation approach;
- Timing of implant placement relative to grafting;
- Duration of follow-up;
- Implant survival rate;
- Number and timing of implant failures;
- Reported causes of implant loss.

Only complications explicitly associated with implant loss were recorded. If a study did not report the cause of implant failure, this was noted as “not specified.” Any discrepancies in data extraction were resolved by consensus.

### 2.7. Risk of Bias Assessment

The methodological quality of included observational studies was assessed using an adapted Newcastle–Ottawa Scale (NOS). Risk of bias was evaluated across the following domains: Representativeness of the exposed cohort; Clarity of inclusion and exclusion criteria; Ascertainment of exposure (documentation of grafting and implant protocol); Control of confounding variables (e.g., cleft type, graft type, implant timing); Assessment of outcomes (clear definition of implant survival and failure criteria); Adequacy and duration of follow-up; Completeness of outcome reporting.

Each domain was judged as low, moderate, or high risk of bias. An overall risk-of-bias judgment for each study was assigned based on the cumulative assessment of domains: Low risk (most domains adequately addressed, prospective design, clear outcome definition, sufficient follow-up); Moderate risk (retrospective design and/or limited control of confounding factors); High risk (unclear outcome definitions, incomplete follow-up reporting, or significant methodological limitations). Disagreements were resolved through discussion and consensus.

### 2.8. Statistical Analysis

A random-effects meta-analysis model was selected due to expected clinical and methodological heterogeneity across studies, including differences in grafting techniques, implant protocols, and follow-up duration. This approach assumes variability in true effect sizes between studies and provides more conservative pooled estimates than fixed-effect models. Logit transformation was applied to stabilize variance when pooling survival proportions, and heterogeneity was assessed using the I^2^ statistic.

Implant survival was analyzed at the implant level. Pooled estimates were calculated using a random-effects meta-analysis of proportions and presented with 95% confidence intervals. Forest plots were constructed to display study-specific and pooled estimates. Prespecified subgroup analyses explored potential sources of heterogeneity related to implant timing and guided bone regeneration (GBR), with differences assessed using the Q test (*p* < 0.05).

For temporal comparisons, studies were categorized into early (1997–2008) and modern (2010–2026) eras. Weighted pooled survival rates were calculated for each subgroup, and differences between proportions were assessed using a two-proportion Z-test. This analysis was considered descriptive and exploratory, as it did not account for inter-study heterogeneity.

## 3. Results

### 3.1. Study Selection

**Figure 1:**
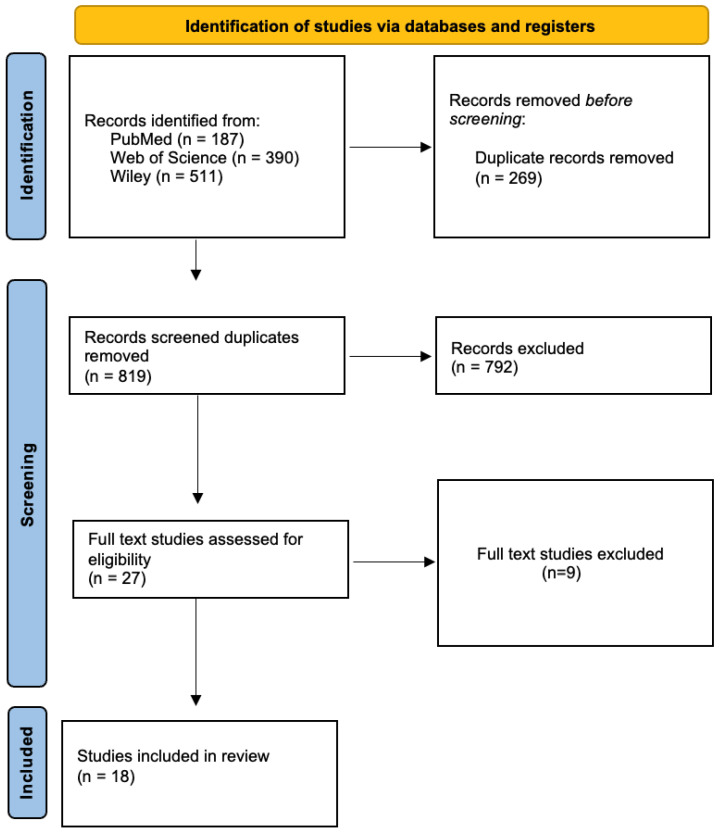
PRISMA flow-chart.

The electronic database search identified 1088 records through the PubMed, Web of Science, Cochrrane Library, and Wiley databases. After the removal of duplicates, 819 unique records remained and were screened based on titles and abstracts. Following this initial screening, 792 records were excluded for not meeting the predefined inclusion criteria. A total of 27 full-text articles were assessed for eligibility. Of these, 9 studies were excluded after full-text review for the following reasons: non-clinical study design, absence of dental implant placement in grafted alveolar cleft sites, lack of implant survival or success outcomes, insufficient follow-up duration, small case series, mixed populations with non-separable cleft data, or outcomes not aligned with the objectives of the review (Table 1). Ultimately, 18 studies fulfilled the inclusion criteria and were included in the qualitative synthesis of this systematic review (Figure 1).

### 3.2. Overall Implant Survival

Across the included studies, implant survival in grafted alveolar cleft sites ranged from 80% to 100%, with the majority of contemporary studies reporting survival rates exceeding 90%. 

**Figure 2:**
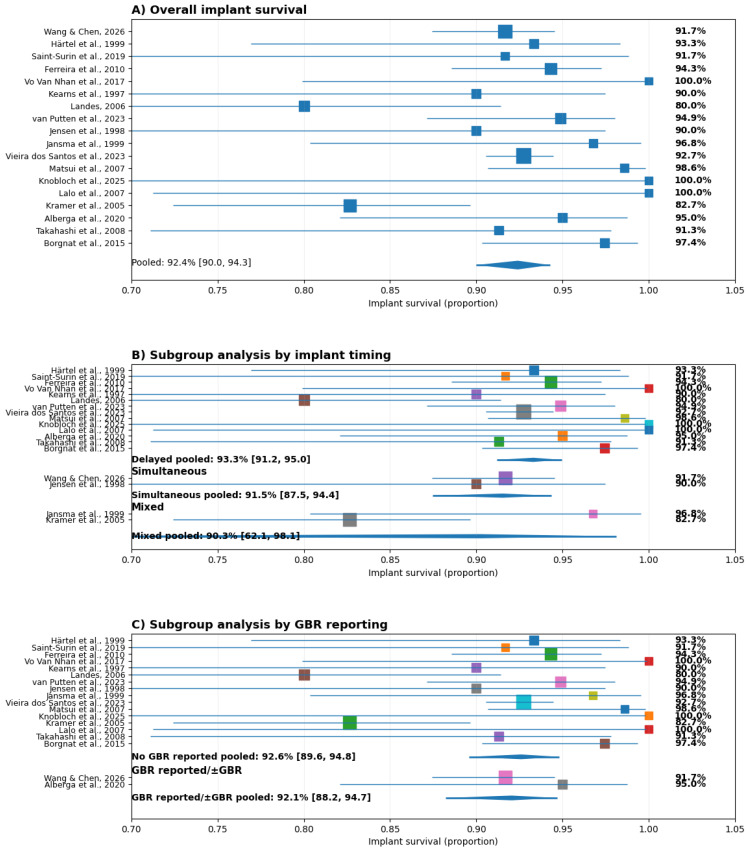
Forest plot analysis of implant survival, implant timing and GBR reporting [21,22,23,24,25,26,27,28,29,30,31,32,33,34,35,36,37,38].

A total of 18 studies were included in the study. Descriptive data extracted from the included studies can be found in (Table 2 and Table 3). The available data was included in the quantitative synthesis of implant survival (Figure 2A). The pooled survival rate was 92.4% (95% CI: 90.0–94.3), indicating a high probability of implant survival across diverse clinical settings and study designs. Individual study estimates ranged from 80.0% to 100%, with most studies reporting survival above 90%. Several investigations demonstrated survival rates of ≥98%, while a limited number reported lower values (80.0–82.7%). Despite this variability, confidence intervals largely overlapped, and the pooled estimate remained stable, suggesting limited heterogeneity and a consistent treatment effect across the included literature.

Early foundational studies from the late 1990s [21,22,23,24] demonstrated survival rates of approximately 90% following delayed implant placement in iliac crest–grafted clefts, with failures predominantly occurring during the early osseointegration phase. Subsequent investigations in the 2000s [25,26,27,28,29,30] reported comparable or improved outcomes, although survival rates as low as 80–82% were observed in cohorts involving short implants, simultaneous placement protocols, or compromised graft volume.

More recent studies (2015–2026) [31,32,33,34,35,36,37,38] consistently demonstrated survival rates between 91% and 100%, reflecting advances in surgical technique, grafting protocols, implant design, and prosthetic planning. Late implant loss was uncommon; most failures occurred within the first year after placement and were attributed to insufficient graft volume, inadequate primary stability, or early osseointegration failure. Peri-implantitis was reported as a cause of late failure in only a limited number of cases (Table 2 and Table 3).

The large retrospective cohort by Vieira dos Santos et al. [35] reported a survival rate of 92.73% across 688 implants, reinforcing the reproducibility of favorable outcomes in cleft populations when appropriate surgical staging is employed. Similarly, morphology-guided augmentation strategies demonstrated survival rates of up to 98.3% at 12 months, suggesting that defect configuration may influence short-term implant stability.

When pooled by era, weighted implant survival increased from 91.2% (95% CI: 87.9–94.5%) in early studies (1997–2008) to 94.2% (95% CI: 92.9–95.5%) in modern studies (2010–2026), representing an absolute difference of 3.0% that reached statistical significance (two-proportion Z-test, *p* = 0.038).

A random-effects meta-analysis confirmed a pooled implant survival rate of 92.4% (95% CI: 90.0–94.3), with moderate heterogeneity across studies (I^2^ = 33.5%) (Figure 2A).

#### 3.2.1. Subgroup Analysis by Implant Timing

Subgroup analysis according to implant placement timing (Figure 2B) showed that delayed placement was the most frequently adopted approach (k = 14; n = 1257) and yielded a pooled survival rate of 93.3% (95% CI: 91.2–95.0). Simultaneous placement was reported in fewer studies (k = 2; n = 260) and demonstrated a pooled survival of 91.5% (95% CI: 87.5–94.4).

Studies categorized as mixed timing showed a pooled survival of 90.3% (95% CI: 62.1–98.1), with wider confidence intervals reflecting the limited number of studies and smaller sample sizes. These studies were not included in direct subgroup comparisons due to methodological heterogeneity.

Overall, the overlapping confidence intervals and similar point estimates indicate that implant survival did not differ significantly between delayed and simultaneous placement strategies. The slightly higher point estimate observed for delayed placement is unlikely to be clinically meaningful.

#### 3.2.2. Subgroup Analysis by GBR Reporting

Subgroup analysis based on the reporting or use of guided bone regeneration (GBR) is presented in Figure 2C. Studies without GBR reporting constituted the majority of the dataset (k = 16) and demonstrated a pooled survival rate of 92.6% (95% CI: 89.6–94.8).

In contrast, studies explicitly reporting GBR use (k = 2) showed a pooled survival of 92.1% (95% CI: 88.2–94.7). The pooled estimates were nearly identical, and the confidence intervals showed substantial overlap, indicating no statistically significant subgroup effect. These findings suggest that the use or reporting of GBR did not materially influence implant survival outcomes in the analyzed studies.

### 3.3. Risk of Bias Assessment

**Figure 3:**
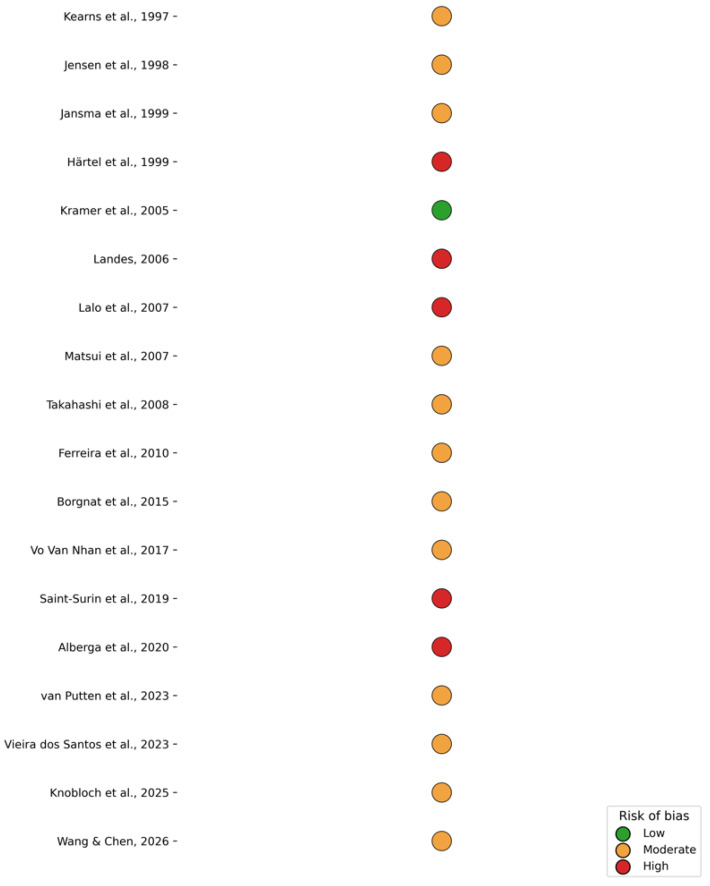
Risk of bias assessment [21,22,23,24,25,26,27,28,29,30,31,32,33,34,35,36,37,38].

According to the Newcastle–Ottawa Scale, most included studies were judged to have a moderate risk of bias, primarily due to retrospective designs and limited adjustment for confounding factors. A small number of case series were classified as high risk, while only one study achieved a low-risk rating. Overall, the methodological quality of the evidence was considered acceptable for observational implant research (Figure 3).

## 4. Discussion

The present systematic review demonstrates a pooled weighted implant survival of 94.2% in the modern era (2010–2026) compared to 91.2% in early-era studies (1997–2008), representing a statistically significant temporal improvement (*p* = 0.038). This temporal trend suggests that implant-supported rehabilitation in grafted alveolar cleft sites has become progressively more predictable over time. The observed improvement likely reflects cumulative advancements in surgical protocols, grafting strategies, implant macro- and microdesign, and interdisciplinary treatment planning [39,40,41,42]. The overall calculated survival rate of 92.4% further confirms the reliability of implant therapy in this anatomically complex population, despite the biological challenges associated with grafted bone and compromised soft tissue architecture.

These findings are broadly consistent with previously published systematic reviews, while providing additional granularity through chronological stratification and explicit evaluation of implant failure timing. Pathak et al. [9] reported an overall calculated implant survival rate of 93.5% irrespective of graft type, closely approximating the survival rate observed in the present analysis and further supporting the consistent clinical performance of implant therapy in grafted cleft sites. However, their meta-analysis did not differentiate outcomes by treatment era nor distinguish early from late implant loss and the inclusion of esthetic and patient-reported outcomes broadened the scope, but limited survival-specific interpretation. Similarly, Sales et al. [5] reported an approximate 93% survival rate across 483 implants with a mean follow-up of 60 months, although they emphasized substantial heterogeneity and the predominance of retrospective designs—limitations that remain evident within the current evidence base.

Wang et al. [3] reported a mean survival of 91.5% ± 4.77% with a mean follow-up of 54 months, closely mirroring the results observed in early-era cohorts in the present review. Notably, Wang et al. highlighted the high frequency of secondary or tertiary grafting, reported in up to 43.1% of cases, underscoring graft stability, maturation, and surgical staging as critical determinants of implant prognosis. In contrast, Wermker et al. [4] reported 5-year survival rates ranging from 80% to 96% (mean 88.6%), lower than contemporary overall survival estimates. They also noted the generally limited methodological quality of available studies—a concern that persists across cleft implant research.

The role of graft maturation and timing is further supported by Mallick et al. [6], who reported success rates between 95% and 100% when tertiary graft healing intervals of 3–6 months were respected. These findings support the biological rationale that controlled healing and sufficient graft consolidation enhance primary stability and osseointegration [42,43]. Conversely, Vuletić et al. [2] provided a descriptive overview of grafting and rehabilitation protocols without an overall calculated implant survival analysis, and Guo et al. [1] focused primarily on secondary bone grafting techniques in pediatric populations without evaluating implant survival, although both emphasized the foundational importance of graft quality prior to implant placement.

Across all reviews, including the present analysis, implant survival in grafted alveolar cleft sites consistently approaches outcomes reported in non-cleft implant populations. Contemporary survival rates exceeding 94% now approximate established implant benchmarks in the broader literature [30,31,32,33,34,35,36,37,38,42]. Nevertheless, survival metrics alone do not fully capture treatment complexity. High rates of tertiary grafting and staged interventions indicate that favorable outcomes are frequently contingent upon additional augmentation procedures and coordinated multidisciplinary care. Implant prognosis in cleft patients should therefore be interpreted within the broader context of surgical burden, orthodontic preparation, and prosthetic planning rather than survival data alone [30,31,32,33,34,35,36,37,38,39,40,44,45,46,47,48,49,50].

The pattern of implant failure identified in this review provides additional clinical insight. Failures occurred predominantly during the early osseointegration phase and were most commonly associated with insufficient graft volume, inadequate primary stability, or early biological complications [51,52]. In contrast, late failures, including peri-implantitis-related loss, were comparatively infrequent. This distribution suggests that implant success in cleft patients is largely determined during the initial healing phase and is more strongly influenced by graft-related and surgical factors than by long-term inflammatory peri-implant breakdown [46,47,48,49,53,54]. Continued maintenance and long-term monitoring are therefore essential in cleft patients to ensure sustained peri-implant health [46,47,48,49,53,54].

Once osseointegration is established, implant behavior in grafted cleft bone appears comparable to that observed in native maxillary bone [42].

Despite encouraging survival rates, the overall evidence base remains methodologically constrained. As noted in previous systematic reviews [3,4,5], most available studies are retrospective case series with a moderate-to-high risk of bias and heterogeneous reporting standards. Differences in follow-up duration, implant systems, grafting techniques, and definitions of survival versus success limit direct inter-study comparability. Earlier reviews reported pooled survival estimates between approximately 88% and 93% without stratification by treatment era or systematic evaluation of failure timing [3,4,5]. By incorporating chronological stratification, weighted implant survival rates and statistical comparison between early and modern cohorts, the present review provides a more nuanced interpretation of temporal improvements. Furthermore, explicit differentiation between early osseointegration failure and late biological complications enhances clinical relevance beyond that of prior analyses.

This review has several strengths. It integrates the recent literature up to 2026, applies a structured methodological framework, and combines quantitative pooling with subgroup and temporal analyses, allowing for a more comprehensive interpretation of implant outcomes in grafted cleft sites. The inclusion of failure timing and causes further enhances clinical relevance by identifying the early healing phase as the most critical period for implant prognosis.

However, several limitations must be acknowledged. The dominance of observational study designs, limited number of prospective investigations, and variability in outcome reporting restrict the strength of causal inference. Subgroup analyses were constrained by imbalances in study numbers, particularly for simultaneous implant placement and GBR reporting, and long-term data beyond 10 years remain scarce. In addition, patient-reported outcomes, esthetic assessments, and functional measures were inconsistently reported, limiting evaluation of treatment success beyond survival. The interpretation of the present findings should consider the overall methodological quality of the included studies. Most evidence derives from retrospective observational designs with moderate risk of bias, particularly related to selection processes, variability in outcome definitions, and limited adjustment for confounding factors. These limitations may influence the precision of pooled estimates and restrict causal inference. Consequently, the reported survival rates should be interpreted as indicative of clinical trends rather than definitive effect estimates.

Future research should prioritize prospective multicenter studies with standardized definitions of implant survival and success, longer follow-up durations, and stratified analysis according to grafting protocols, implant timing, and defect morphology. Such studies are essential to refine clinical guidelines and further optimize implant-supported rehabilitation strategies in cleft populations.

## 5. Conclusions

Implant-supported rehabilitation in grafted alveolar cleft sites shows favorable survival outcomes in contemporary clinical reports. Based on predominantly observational evidence, most failures occur during early osseointegration and are commonly associated with insufficient graft volume or limited primary stability.

When adequate bone reconstruction and interdisciplinary planning are achieved, implant therapy appears to be a viable option within comprehensive cleft rehabilitation. However, higher-level prospective studies with standardized protocols and long-term follow-up are needed to strengthen the evidence base and refine clinical recommendations.

## Figures and Tables

**Table 1:** Excluded full-text studies by reason.

Study	Year	Reason for Exclusion
Esper et al. [12]	2012	No implant survival or success outcomes
Dušková et al. [13]	2007	No implant survival or success outcomes
Dempf et al. [14]	2002	No implant survival or success outcomes
Filho et al. [15]	2013	Outcome focus outside review scope
Sogancı et al. [16]	2016	Non-clinical (finite element analysis)
Dowgierd et al. [17]	2021	Non-comparable intervention (free-flap reconstruction)
Green & Padwa [18]	2021	No implant survival or success outcomes
Green et al. [19]	2024	Primary focus on bone augmentation
Cune et al. [20]	2004	Case series with fewer than 10 patients

**Table 2:** Implant survival rates in grafted alveolar cleft sites.

Study (Year)	Study Design	Patients (n)	Implants (n)	Cleft Type	Graft/ Augmentation	Implant Timing	Follow-Up (Mean)	ISR
Kearns et al., 1997 [21]	Prospective cohort	14	20	CLP	Iliac crest Cancellous bone graft	Delayed	39.1 months	90%
Jensen et al., 1998 [22]	Prospective case series	16	20	Residual alveolar Cleft	Mandibular symphyseal bone graft	Simultaneous	48 months	90%
Jansma et al., 1999 [23]	Prospective case series	15	31	CLP	Iliac crest/ Mandibular symphysis grafts	Simultaneous/Delayed	47–56 months	96.8%
Härtel et al., 1999 [24]	Retrospective case series	14	30	CLP	Autogenous bone graft	Delayed	≥24 months	≈93%
Kramer et al., 2005 [25]	Prospective cohort	45	75	CLAP	Iliac crest graft (Tertiary)	Simultaneous/Delayed	5.5 years	82.2%
Landes, 2006 [26]	Retrospective comparative	20	25	CLAP	Secondary/tertiary grafts	Delayed	44 months	≈80%
Lalo et al., 2007 [27]	Retrospective case series	12	20	CLP	Autogenous bone graft	Delayed	5.5 years	100%
Matsui et al., 2007 [28]	Retrospective cohort	47	71	CLP	Iliac crest PCBM	Delayed	60 months	98.6%
Takahashi et al., 2008 [29]	Longitudinal cohort	16	23	CLP	Iliac crest PCBM	Delayed	8.6 years	90.9%
Ferreira et al., 2010 [30]	Retrospective cohort	120	123	Uni/ Bilateral CLP	Iliac crest graft	Delayed	34 months	94.3%
Borgnat et al., 2015 [31]	Retrospective cohort	43	78	CLP	Autogenous bone	Delayed	Up to 15 years	97.4%
Vo Van Nhan et al., 2017 [32]	Prospective case series	32	32	CLP	Iliac crest graft	Delayed	18–53 months	100%
Saint-Surin et al., 2019 [33]	Retrospective cohort	39	12	Alveolar cleft	Complementary alveolar bone grafting	Delayed	27 months	91.7%
Alberga et al., 2020 [34]	Retrospective comparative	27	40	CLP	Iliac crest graft ± GBR	Delayed	Mean 72.4 mo	95.0%
van Putten et al., 2023 [35]	Retrospective cohort	64	78	CLA/CLAP	Secondary/tertiary grafts	Delayed	46 months	95.0%
Vieira dos Santos et al., 2023 [36]	Retrospective cross-sectional	—	688	CLP	Grafted cleft sites ± regrafting	Delayed	53.2 months	92.73%
Knobloch et al., 2025 [37]	Prospective cohort	14	17	CP	Surgically corrected cleft site	Delayed	5 years	100%
Wang & Chen, 2026 [38]	Retrospective comparative	240	240	CLP	Iliac crest + GBR	Simultaneous	12 months	91.7–98.3%

ISR = implant survival rate. CLP = cleft lip and palate. CLA = cleft lip and alveolus. CLAP = cleft lip, alveolus, and palate. CP = cleft palate. GBR = guided bone regeneration. PCBM = particulate cancellous bone and marrow harvested from the iliac crest.

**Table 3:** Implant loss, timing, implant system, and causes of failure in grafted alveolar cleft sites.

Study (Year)	Implant System	Diameter/Length (mm)	Implants Placed (n)	Implants Lost (n)	Timing of Loss	Reported Cause of Implant Loss
Kearns et al., 1997 [21]	Brånemark	NR	20	2	Early	Insufficient grafted bone volume; Early osseointegration implant failure
Jensen et al., 1998 [22]	Brånemark	NR	20	2	Early	Graft sequestration Wound dehiscence
Jansma et al., 1999 [23]	Brånemark	NR	31	1	Early	Early osseointegration implant failure
Härtel et al., 1999 [24]	Brånemark	NR	30	2	Early	Insufficient bone volume; Early osseointegration failure
Kramer et al., 2005 [25]	NR	<13 mm	75	10	Early (≤1 years)	Short implant length Early osseointegration failure
Landes, 2006 [26]	NR	NR	25	5	Early & late	Short implants Osseointegration failure
Lalo et al., 2007 [27]	NR	NR	20	0	NR	NR
Matsui et al., 2007 [28]	Brånemark	13–15 mm (length)	71	1	Early	Osseointegration failure
Takahashi et al., 2008 [29]	Brånemark	NR	23	2	Early	Insufficient grafted bone volume
Ferreira et al., 2010 [30]	NR	NR	123	7	Early	Insufficient grafted bone volume
Borgnat et al., 2015 [31]	NR	NR	78	2	Early	Poor bone quality; Early osseointegration failure
Vo Van Nhan et al., 2017 [32]	NR	NR	32	0	NR	NR
Saint-Surin et al., 2019 [33]	NR	NR	12	1	Early	Early osseointegration failure
Alberga et al., 2020 [34]	Straumann; Nobel Biocare	3.3–4.1 mm (diameter)	40	2	Early	Lack of primary stability
van Putten et al., 2023 [35]	NR	NR	78	5	Early (n = 2); Late (n = 3)	Early: inadequate osseointegration; Late: peri-implantitis
Vieira dos Santos et al., 2023 [36]	NR	NR	688	50 *	NR	NR
Knobloch et al., 2025 [37]	AstraTech OsseoSpeed	3.0–3.5 mm (diameter)	17	0	—	NR
Wang & Chen, 2026 [38]	NR	NR	240	5	≤12 months	Graft instability; Implant failure

NR = Not Reported. * Failure rate 7.27% reported; absolute number estimated from provided data.

## Data Availability

The original contributions presented in this study are included in the article/Supplementary Materials. Further inquiries can be directed to the corresponding author(s).

## Supplementary Materials

The following supporting information can be downloaded at https://www.mdpi.com/article/10.3390/medicina62030569/s1. Table S1: PRISMA 2020 Checklist.
